# Supplementary material for: Comparative genomics provides new insights into the diversity, physiology, and sexuality of the only industrially exploited tremellomycete: Phaffia rhodozyma
Source: BMC Genomics. 2016 Nov 9;17:901. doi: 10.1186/s12864-016-3244-7 (PMC5103461; doi:10.1186/s12864-016-3244-7)
Supplement: Additional file 6: — List of orphan genes with links to PFAM (related to Additional file 1: Table S1). (ZIP 1428 kb) [file 12864_2016_3244_MOESM6_ESM.zip › PFAM_UNIQUE/G04047_P.html]

#                                                                            --- full sequence --- -------------- this domain -------------   hmm coord   ali coord   env coord
  
# target name        accession   tlen query name           accession   qlen   E-value  score  bias   #  of  c-Evalue  i-Evalue  score  bias  from    to  from    to  from    to  acc description of target
  
#------------------- ---------- ----- -------------------- ---------- ----- --------- ------ ----- --- --- --------- --------- ------ ----- ----- ----- ----- ----- ----- ----- ---- ---------------------
  
G04047\_P             -            312 Bacteriocin\_IIc      PF10439.4     65       4.7    6.1  27.5   1   3     0.017        17    4.3   3.7    27    60    54    87    35    90 0.72 - Bacteriocin class II with double-glycine leader peptide  
G04047\_P             -            312 Bacteriocin\_IIc      PF10439.4     65       4.7    6.1  27.5   2   3   0.00085      0.85    8.5   4.2    17    52   107   141   104   145 0.85 - Bacteriocin class II with double-glycine leader peptide  
G04047\_P             -            312 Bacteriocin\_IIc      PF10439.4     65       4.7    6.1  27.5   3   3     0.015        15    4.5   3.9    31    58   259   285   251   291 0.47 - Bacteriocin class II with double-glycine leader peptide
